# Impaired Interoceptive Accuracy in Semantic Variant Primary Progressive Aphasia

**DOI:** 10.3389/fneur.2017.00610

**Published:** 2017-11-16

**Authors:** Charles R. Marshall, Chris J. D. Hardy, Lucy L. Russell, Camilla N. Clark, Katrina M. Dick, Emilie V. Brotherhood, Rebecca L. Bond, Catherine J. Mummery, Jonathan M. Schott, Jonathan D. Rohrer, James M. Kilner, Jason D. Warren

**Affiliations:** ^1^Dementia Research Centre, Department of Neurodegenerative Disease, London, United Kingdom; ^2^Sobell Department of Motor Neuroscience and Movement Disorders, Institute of Neurology, University College London, London, United Kingdom

**Keywords:** interoception, autonomic, cardiac, empathy, primary progressive aphasia, frontotemporal dementia

## Abstract

**Background:**

Interoception (the perception of internal bodily sensations) is strongly linked to emotional experience and sensitivity to the emotions of others in healthy subjects. Interoceptive impairment may contribute to the profound socioemotional symptoms that characterize frontotemporal dementia (FTD) syndromes, but remains poorly defined.

**Methods:**

Patients representing all major FTD syndromes and healthy age-matched controls performed a heartbeat counting task as a measure of interoceptive accuracy. In addition, patients had volumetric MRI for voxel-based morphometric analysis, and their caregivers completed a questionnaire assessing patients’ daily-life sensitivity to the emotions of others.

**Results:**

Interoceptive accuracy was impaired in patients with semantic variant primary progressive aphasia relative to healthy age-matched individuals, but not in behavioral variant frontotemporal dementia and nonfluent variant primary progressive aphasia. Impaired interoceptive accuracy correlated with reduced daily-life emotional sensitivity across the patient cohort, and with atrophy of right insula, cingulate, and amygdala on voxel-based morphometry in the impaired semantic variant group, delineating a network previously shown to support interoceptive processing in the healthy brain.

**Conclusion:**

Interoception is a promising novel paradigm for defining mechanisms of reduced emotional reactivity, empathy, and self-awareness in neurodegenerative syndromes and may yield objective measures for these complex symptoms.

## Introduction

Interoception (the ability to sense one’s internal physiological states) is closely linked to emotional experience (1) and can be measured using awareness of one’s heartbeat as a surrogate for interoceptive sensitivity (2, 3). According to recent interoceptive inference formulations, hierarchically organized brain networks compare afferent interoceptive information with predictions about bodily states, with prediction errors activating autonomic reflexes or motivating actions to maintain homeostasis (4). At lower hierarchical levels, these relate to direct physiological homeostasis, such as maintaining blood oxygen and glucose levels. Coherent representations of the physiological state of one’s body are important determinants of subjective feeling states (5), and those with weaker interoception are less able to identify and describe their own emotions (6). At higher hierarchical levels, inferences about more complex causes of physiological perturbations can be made, such as the autonomic changes induced by the emotions of others. Interoception is therefore hypothesized to play a key role in empathy and theory of mind (7). This is borne out by evidence showing that interoceptive ability predicts both sensitivity to the emotions of others and performance on emotional theory of mind tasks (8, 9). Empathy has been correlated with the magnitude of heartbeat-evoked potentials, and both cognitive and neural responses to the emotions of others are influenced by stimulus timing within the cardiac cycle (10–12).

Interoceptive signals and exteroceptive information from the environment are integrated in a reciprocal manner, with diminished interoception tending to promote greater environmental dependency, and *vice versa*. Those with less interoceptive ability are more susceptible to exteroceptive signals that alter perception of body ownership (13), while inducing the illusion of decreased body ownership reduces both the amplitude of heartbeat-evoked potentials (14) and the ability to cognitively detect signals arising from the heart (15). Interoception is therefore likely to play a key role in generating a coherent sense of the bodily self. The reciprocal relationship between interoception and exteroception has also been demonstrated in perceptual decision-making, with interoceptive arousal limiting the influence of exteroceptive sensory noise on confidence (16). Interoception entails dissociable cognitive dimensions, interoceptive accuracy (objective reporting) supporting awareness (confidence in interoceptive judgments) (3). Interoceptive sensitivity is mediated principally by cingulate and insula (17) under the influence of amygdala (18). Together, these structures constitute a network engaged in both interoception and emotion processing (5).

Interoception has been hypothesized to be a factor mediating changes in emotional sensitivity in normal aging (19). Different dimensions of interoception—accuracy and awareness—might be separately targeted by brain disease. One leading candidate, on clinical and neuroanatomical grounds, is the group of neurodegenerative diseases comprising frontotemporal dementia (FTD). This heterogeneous entity comprises three major clinico-anatomical syndromes: behavioral variant frontotemporal dementia (bvFTD), semantic variant primary progressive aphasia (svPPA), and nonfluent variant primary progressive aphasia (nfvPPA). All three syndromes profoundly disrupt emotional and physiological reactivity (20–22), producing complex neuropsychiatric symptoms such as loss of empathy and altered bodily awareness (23, 24). These symptoms are of key clinical relevance but remain difficult to measure and poorly understood (25). Impaired interoception is a plausible mechanism that may link neurodegeneration to socioemotional phenotypes in FTD (26). However, interoceptive processing has not been studied systematically in the FTD syndromic spectrum nor specifically related to reduced emotional awareness in particular FTD syndromes and to underlying neuroanatomical substrates (26).

Here, we used heartbeat counting to assess interoceptive accuracy in canonical FTD syndromes (svPPA, bvFTD, and nfvPPA) versus healthy older individuals. We related patients’ interoceptive accuracy both to a clinical index of emotional sensitivity and to regional gray matter on voxel-based morphometry (VBM). As all syndromes within the FTD spectrum are associated with socioemotional deficits and insular atrophy, some degree of impaired interoception leading to abnormal emotional awareness is anticipated across the FTD spectrum. However, among FTD syndromes, svPPA in particular has been linked to abnormally heightened responsiveness to exteroceptive stimuli (24), altered bodily awareness, and an impoverished concept of self (27). The associations between interoception, exteroception, body ownership, and sense of self identified in the healthy brain (13–16) suggest that reduced interoceptive accuracy may be a core feature of svPPA and disproportionately severe in this syndrome relative to other FTD syndromes. Moreover, incorporation of interoceptive information into emotional judgments has been shown to depend on the amygdala, which is particularly severely affected in svPPA (18, 28). This further suggests a brain mechanism that could link reduced interoceptive accuracy to loss of emotional sensitivity in this syndrome. We therefore hypothesized that all FTD syndromes would be associated with a degree of impaired interoception leading to reduced emotional sensitivity, but that svPPA would be associated with a particularly severe deficit of interoceptive accuracy, based on the specific psychophysiological profile of this syndrome and linked to grey matter loss in a frontotemporal network including amygdala.

## Materials and Methods

### Participants

Thirty-two consecutive patients fulfilling consensus criteria for a syndrome of FTD (29, 30) (16 bvFTD, 7 svPPA, and 9 nfvPPA) and 19 age-matched healthy individuals [overall 51 participants, mean age 67.6 years (range 51–84), 22 females] participated. No participant had a history of cardiac arrhythmia, clinical depression, or anxiety disorder. Neuropsychological assessment and MR brain imaging corroborated the syndromic diagnosis in all patients. Clinical, demographic, and neuropsychological characteristics of all participants are summarized in Table 1. Participant groups did not differ significantly in age or gender, symptom duration, or use of antihypertensive medication; no participant was taking cardiac rate-limiting medication. The study was approved by the local ethics committee and all participants gave informed consent.

**Table 1:** Clinical and neuropsychological characteristics of participant groups.

Characteristic	Controls	bvFTD	svPPA	nfvPPA
**Demographic and clinical**				
No (m:f)	8:11	13:3	5:2	4:5
Age (years)	68.8 (5.5)	65.8 (7.3)	65.9 (7.4)	69.6 (6.5)
Handedness (R:L:A)	17:1:1	15:1:0	7:0:0	7:2:0
MMSE (/30)	29.6 (0.6)	24.6 (4.5)^a^	22.6 (5.8)^a^	23.7 (6.0)^a^
Duration (years)	N/A	7.6 (4.7)	4.4 (2.0)	4.6 (2.2)
EX	N/A	5.4 (4.7)^d^	9.5 (2.3)^d^	20.0 (7.6)
Mean heart rate	69.5 (10.2)^d^	72.5 (12.9)	69.7 (5.2)^d^	85.5 (17.1)
**Neuropsychological**				
General intellect				
WASI verbal IQ	125.4 (7.0)	86.4 (22.4)^a^	78.6 (20.4)^a^	80.0 (17.3)^a^
WASI performance IQ	125.1 (9.7)	102.44 (21.4)^a^	112.3 (20.1)	98.8 (21.5)^a^
**Episodic memory**				
RMT words (/50)	49.3 (0.9)	36.2 (8.0)^a^	30.3 (6.9)^a,d^	41.4 (9.5)^a^
RMT faces (/50)	44.7 (3.7)	34.0 (7.6)^a^	32.7 (6.4)^a^	39.5 (6.6)
Camden PAL (/24)	20.3 (3.5)	10.5 (7.5)^a^	2.7 (4.2)^a,b,d^	16.3 (7.8)
**Executive skills**				
WASI block design (/71)	46.0 (10.1)	32.6 (19.2)	41.6 (19.0)	25.1 (19.7)^a^
WASI matrices (/32)	26.6 (4.1)	17.8 (9.4)^a^	21.7 (8.5)	17.4 (9.0)^a^
WMS-R digit span forward (max)	7.1 (1.2)	6.6 (1.2)	7.0 (1.2)	4.8 (0.8)^a,b,c^
WMS-R digit span reverse (max)	5.6 (1.3)	4.4 (1.4)	5.1 (2.0)	3.0 (0.7)^a^
D-KEFS Stroop color naming (s)	32.4 (6.4)^b,d^	49.5 (20.8)^d^	50.3 (27.9)^d^	87.0 (6.7)
D-KEFS Stroop word reading (s)	23.5 (5.7)^d^	35.9 (22.2)^d^	30.9 (19.2)^d^	85.4 (10.3)
D-KEFS Stroop interference (s)	56.2 (16.9)^b,d^	103.3 (47.3)^d^	82.7 (50.5)^d^	165.0 (30.8)
Letter fluency (F: total)	18.1 (5.7)	7.6 (4.4)^a^	9.7 (7.2)^a^	3.5 (1.7)^a^
Category fluency (animals: total)	24.7 (5.9)	11.6 (6.2)^a^	6.7 (5.4)^a^	8.8 (3.5)^a^
Trails A (s)	32.2 (5.6)^b,d^	59.5 (33.5)	47.0 (21.0)	81.7 (48.4)
Trails B (s)	66.1 (20.5)^b,d^	184.1 (89.0)	133.6 (110.1)	211.1 (94.6)
**Language skills**				
WASI vocabulary	72.2 (3.4)	42.6 (21.8)^a^	34.7 (22.7)^a^	31.7 (13.9)^a^
BPVS	148.5 (1.1)	123.8 (35.3)^a^	94.4 (49.4)^a,d^	142.6 (10.1)
GNT	26.3 (2.4)	10.6 (9.8)^a^	2.0 (5.3)^a,b,d^	15.5 (6.6)^a^
**Posterior cortical skills**				
GDA (/24)	15.8 (5.4)	7.8 (5.7)^a^	11.3 (8.3)	5.4 (1.9)^a^
VOSP Object Decision (/20)	19.1 (1.6)	15.6 (3.0)^a^	15.7 (5.1)	15.3 (4.7)^a^

*Mean (SD) scores are shown unless otherwise indicated; maximum scores are shown after tests (in parentheses)*.

*^a^Significantly less than controls, ^b^significantly less than bvFTD, ^c^significantly less than SD, ^d^significantly less than PNFA (all *p* < 0.05)*.

*BPVS, British Picture Vocabulary Scale (31); category fluency for animal category and letter fluency for the letter F in 1 min (32); EX, sensitivity to the emotions of others component of the Revised Self-Monitoring Scale (33); GDA, Graded Difficulty Arithmetic (34); GNT, Graded Naming Test (35); MMSE, Mini-Mental State Examination score (36); N/A, not assessed; PAL, Paired Associate Learning test (37); RMT, Recognition Memory Test (38); Stroop D-KEFS, Delis Kaplan Executive System (39); Trails-making task based on maximum time achievable 2.5 min on task A, 5 min on task B (40); VOSP, Visual Object and Spatial Perception Battery (41); WAIS-R, Wechsler Adult Intelligence Scale – Revised (42); WASI, Wechsler Abbreviated Scale of Intelligence (43); WMS, Wechsler Memory Scale (44)*.

### Heartbeat Counting Task

We adapted a previously described heartbeat counting task as a measure of interoceptive accuracy (2, 3). Participants were asked to try to identify their heartbeats by “listening to their body” (rather than feeling their pulse) and were first familiarized with the paradigm to ensure they understood the task. ECG was recorded continuously from electrodes placed over the right clavicle and left iliac crest. During the experiment, the number of sensed beats was reported for four epochs of variable duration (25, 35, 45, and 100 s) signaled by start and stop tones and presented in randomized order, to preclude anticipation or guessing based on previous epochs. For each participant, an interoceptive accuracy index (IA) was calculated based on an established method as follows (3):

$$\begin{array}{l} 1\;-\;\left|\text{actual beats }-\text{ reported beats}\right|\;/((\text{actual beats}+\text{reported beats})/2). \end{array}$$

### Emotional Sensitivity Rating

Patients’ caregivers completed the Sensitivity to Socioemotional Expressiveness Score (EX) component of the Revised Self-Monitoring Scale (33), a daily-life index of sensitivity to the emotions of others.

### Data Analysis

Between-group differences were assessed using ANOVAs, except where the homogeneity of variance assumption was violated, when Welch’s *F* test and Games Howell *post hoc* tests (a multiple comparison procedure without the assumption of homoscedasticity) were used. In addition, we assessed correlations of IA with EX (sensitivity to others’ emotions), auditory reverse digit span (a standard index of nonverbal sensory working memory), British Picture Vocabulary score (a standard measure of semantic comprehension), and mean heart rate (a peripheral interoceptive signal characteristic). A threshold *p* < 0.05 was accepted as the significance criterion for all tests.

### Brain Image Acquisition and Analysis

Each patient had a sagittal 3-D magnetization-prepared rapid-gradient-echo T1-weighted volumetric brain MR sequence (TE/TR/TI 2.9/2,200/900 ms, dimensions 256 256 208, voxel size 1.1 mm^3^), acquired on a Siemens Trio 3 T MRI scanner using a 32-channel phased-array head-coil. Normalization, segmentation, and modulation of gray and white matter images were performed using SPM12^1^ with default parameter settings and gray matter images were smoothed using a 6 mm full width-at-half-maximum Gaussian kernel. A study-specific template mean brain image was created by warping all bias-corrected native space brain images to the final DARTEL template and calculating the average of the warped brain images. Total intracranial volume (TIV) was calculated for each patient by summing gray matter, white matter, and cerebrospinal fluid volumes following segmentation of all three tissue classes.

A full factorial model was used to assess associations between IA and regional gray matter volume (voxel intensity) within each syndromic group, incorporating age and TIV as covariates of no interest. Statistical parametric maps of regional gray matter associations were assessed at threshold *p* < 0.05 after family-wise error (FWE) correction for multiple voxel-wise comparisons within pre-determined regions of interest [cingulate cortex, insula, and amygdala (17, 18) defined from the Harvard-Oxford Brain Atlas].^2^

## Results

**Figure 1:**
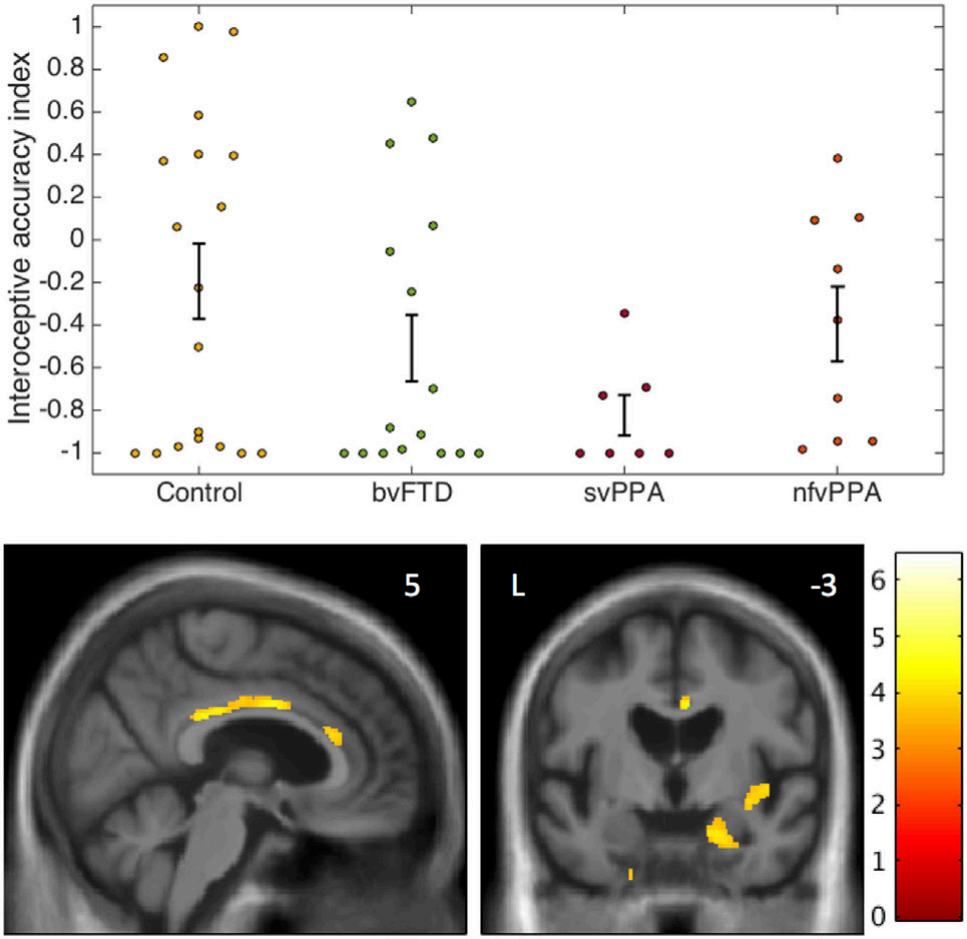
Interoceptive accuracy in participant groups: behavioral and voxel-based morphometry data. The plots (above) show individual raw data for accuracy on the heartbeat counting task expressed as an interoceptive accuracy index (see text) in each participant group. Error bars represent SEM. The statistical parametric map of regional gray matter volume associated with interoceptive accuracy in the impaired svPPA group (below) has been overlaid on representative sections of the normalized study-specific T1-weighted group mean brain MR image; the MNI coordinate (millimeters) of the plane of each section is indicated. The color barcodes *T* values; the SPM is thresholded here at *p* < 0.001 uncorrected over the whole brain for display purpose. Regional local maxima were significant at *p* < 0.05_FWE_ corrected for multiple comparisons over the whole brain (right amygdala, MNI coordinates [18 −15 −21]) or within pre-specified anatomical regions of interest (anterior cingulate cortex [4 0 34]; posterior cingulate cortex [2 −30 28]; right insula [44 −4 −3]). bvFTD, patients with behavioral variant frontotemporal dementia; Control, healthy control group; L, left; nfvPPA, patients with nonfluent variant primary progressive aphasia; svPPA, patients with semantic variant primary progressive aphasia.

Interoceptive accuracy data and neuroanatomical correlates are presented in Figure 1.

The homogeneity of variance assumption was violated for IA data (Levene’s test *p* = 0.001). Welch’s *F* test revealed a main effect of participant group on IA (*p* = 0.021). Games Howell *post hoc* tests showed that IA was significantly lower in the svPPA group than healthy controls (*p* = 0.022). No other significant group differences were identified for IA. Mean EX was significantly higher in the nfvPPA group than the other patient groups (*p* < 0.001) but did not differ between the bvFTD and svPPA groups (*p* = 0.29). Across the patient cohort, there was a significant positive correlation between IA and EX (rho = 0.516, *p* = 0.004); there was no significant association between IA and reverse digit span (rho = 0.133, *p* = 0.372), British Picture Vocabulary Score (rho = 0.242, *p* = 0.09), mean heart rate (rho = 0.038, *p* = 0.8), age (rho = −0.062, *p* = 0.67), disease duration (rho = −0.1, *p* = 0.59), or antihypertensive use (*p* = 0.5).

In the svPPA group, IA was significantly positively associated with gray matter volume in right amygdala, right anterior, and posterior cingulate cortex and right insula (all *p* < 0.05_FWE_ within pre-specified regions of interest). No significant gray matter associations were identified at the prescribed threshold in the other patient groups.

## Discussion

Our findings demonstrate that interoceptive accuracy is impaired in svPPA relative to healthy older individuals. There was a wide range of IA scores in the control group, as typically found in studies of healthy individuals (3, 13). Overall performance in the control group was lower than typically found in studies of younger subjects, with several being unable to detect heartbeats, but this is consistent with evidence that interoception declines with age (45–47). Over the patient cohort, impaired IA did not correlate with any reduction in generic sensory monitoring, semantic capacity, or peripheral interoceptive signal. In line with current models of interoception (3, 17, 18) and evidence for abnormal processing of homeostatic and affective signals in FTD syndromes (21), the findings suggest that svPPA affects the initial cognitive decoding of interoceptive signals. Interoceptive accuracy in the patient cohort was correlated with sensitivity to others’ emotions: coupled with evidence in the healthy brain (3, 8–10, 17, 18), this suggests that degraded inference of others’ emotions from one’s own embodied responses might serve as a generic mechanism for the blunted emotional reactivity and empathy loss that characterizes FTD and may be particularly pervasive in svPPA (20). Moreover, interoceptive impairment is a plausible mechanism for the severe impoverishment of self-projection described in svPPA, and for the increased dependency on exteroceptive signals found in these patients (24, 27).

Emotional sensitivity was comparably reduced in both the svPPA and bvFTD groups (relative to the nfvPPA group) here, while bvFTD has been associated with impaired interoceptive awareness in previous work (26). Taken together with the present findings, the emerging picture suggests a complex stratification of autonomic abnormalities across FTD syndromes: autonomic reactivity in these syndromes may be differentially altered under particular conditions (such as detection of salient changes in self or environment versus monitoring of bodily states) (21, 26). FTD syndromes may target separable levels of interoceptive processing, svPPA producing a more fundamental deficit of interoceptive signal analysis and decoding of autonomic responses to emotion, while bvFTD impairs autonomic reactivity and the metacognitive analysis of body state representations in self and others (22, 48, 49). The neuroanatomical substrate for impaired interoceptive accuracy in the present svPPA group comprised a rightward-asymmetric cingulo-insulo-amygdalar network: this network is encompassed by the distributed atrophy profile of svPPA (28), has been previously implicated in interoception both in the healthy brain and disease states (17, 18, 26), and is well-placed anatomically to integrate homeostatic and external socioemotional signals in building representations of self and others (5).

This small study provides proof of principle for further systematic investigation of interoception as an attractive, novel paradigm for deconstructing complex deficits of emotional reactivity, empathy, and self-awareness in neurodegenerative syndromes. At present, we lack quantifiable metrics for cardinal socioemotional symptoms of dementia. Interoception may plausibly underpin such symptoms and can be assessed using simple, objectively verifiable procedures. Clearly, the variation in intrinsic interoceptive sensitivity among healthy people will need to be taken into account in applying interoceptive measures in clinical settings. However, acknowledging this caveat, interoceptive sensitivity warrants further evaluation, both as a potential biomarker in individuals with retained baseline capacity to perform the task and to identify neuroanatomical and physiological correlates, which might yield outcome measures in clinical trials. Future work should assess different interoceptive dimensions longitudinally, in larger cohorts sampling representatively across syndromes and with molecular correlation, to determine the reliability, sensitivity, and specificity of potential interoceptive biomarkers. Larger studies with greater power may additionally reveal less profound interoceptive deficits within the heterogeneous bvFTD population. Control conditions involving exteroceptive counting tasks of comparable difficulty might help to further disambiguate interoceptive deficits from other cognitive difficulties impairing task performance. The use of passive interoception tasks such as those based on stimulus timing in the cardiac cycle and measurement of heartbeat-evoked potentials would also be of value to provide further confirmation that deficits in interoceptive reporting are not confounded by other neuropsychological impairments (12, 50).

## Ethics Statement

This study was carried out in accordance with the recommendations of The National Hospital for Neurology and Neurosurgery and Institute of Neurology Joint Research Ethics Committee with written informed consent from all subjects. All subjects gave written informed consent in accordance with the Declaration of Helsinki. The protocol was approved by The National Hospital for Neurology and Neurosurgery and Institute of Neurology Joint Research Ethics Committee.

## Author Contributions

Conception and design of the study: CM, JK, and JW. Acquisition and analysis of data: CM, CH, LR, CC, KD, EB, CM, JS, JR, JK, and JW. Drafting of manuscript: CM, CH, RB, JS, JK, and JW.

## Conflict of Interest Statement

The authors declare that the research was conducted in the absence of any commercial or financial relationships that could be construed as a potential conflict of interest.

## Footnotes

^1^www.fil.ion.ucl.ac.uk/spm.

^2^http://fsl.fmrib.ox.ac.uk/fsl/fslwiki/Atlases.
